# Intracranial Pressure Dysfunction Following Severe Intracerebral Hemorrhage in Middle-Aged Rats

**DOI:** 10.1007/s12975-022-01102-8

**Published:** 2022-11-11

**Authors:** Anna C. J. Kalisvaart, Ashley H. Abrahart, Alyvia T. Coney, Sherry Gu, Frederick Colbourne

**Affiliations:** 1 Department of Psychology, University of Alberta, Edmonton, AB Canada; 2 Neuroscience and Mental Health Institute, University of Alberta, Edmonton, AB Canada

**Keywords:** Stroke, Intracerebral hemorrhage, Edema, Cell volume, Aging, Intracranial pressure

## Abstract

Rising intracranial pressure (ICP) aggravates secondary injury and heightens risk of death following intracerebral hemorrhage (ICH). Long-recognized compensatory mechanisms that lower ICP include reduced cerebrospinal fluid and venous blood volumes. Recently, we identified another compensatory mechanism in severe stroke, a decrease in cerebral parenchymal volume via widespread reductions in cell volume and extracellular space (tissue compliance). Here, we examined how age affects tissue compliance and ICP dynamics after severe ICH in rats (collagenase model). A planned comparison to historical young animal data revealed that aged SHAMs (no stroke) had significant cerebral atrophy (9% reduction, *p* ≤ 0.05), ventricular enlargement (9% increase, *p* ≤ 0.05), and smaller CA1 neuron volumes (21%, *p* ≤ 0.05). After ICH in aged animals, contralateral striatal neuron density and CA1 astrocyte density significantly increased (12% for neurons, 7% for astrocytes, *p* ≤ 0.05 vs. aged SHAMs). Unlike young animals, other regions in aged animals did not display significantly reduced cell soma volume despite a few trends. Nonetheless, overall contralateral hemisphere volume was 10% smaller in aged ICH animals compared to aged SHAMs (*p* ≤ 0.05). This age-dependent pattern of tissue compliance is not due to absent ICH-associated mass effect (83.2 mm^3^ avg. bleed volume) as aged ICH animals had significantly elevated mean and peak ICP (*p* ≤ 0.01), occurrence of ICP spiking events, as well as bilateral evidence of edema (e.g., 3% in injured brain, *p* ≤ 0.05 vs. aged SHAMs). Therefore, intracranial compliance reserve changes with age; after ICH, these and other age-related changes may cause greater fluctuation from baseline, increasing the chance of adverse outcomes like mortality.

**Supplementary Information:**

The online version contains supplementary material available at 10.1007/s12975-022-01102-8.

## Introduction

Elevated intracranial pressure poses a potentially fatal threat following serious brain injuries or stroke, such as intracerebral hemorrhage (ICH). This occurs when ruptured cerebral vasculature bleeds into brain parenchyma, adding to cranial mass as the hematoma forms. In the days following ICH, vasogenic edema develops, straining the limited cranial capacity and increasing intracranial pressure (ICP) [1]. In the worst cases, there is a vicious cycle of impaired cerebral blood flow fueling cell death and edema, leading eventually to brainstem herniation and death [2]. Even in milder cases, there is widespread neuronal dysfunction owing to diverse perturbations (e.g., ionic dyshomeostasis surrounding the hematoma) [3]. Several physiological responses are engaged to combat rising ICP resulting from mass effect: principally, cerebrospinal fluid (CSF) and venous blood are displaced from the cephalic region [4]. This dynamic relationship between blood, CSF, and brain tissue within the cranium was outlined by Monro and Kellie in 1783, defining ICP as the sum of the pressures of these three components [2]. However, they maintained that as blood and CSF are redirected from the cranium to maintain ICP homoeostasis, the volume of brain parenchyma remains constant, with “low compressibility”; this view has persisted for centuries [2].

Although measuring contribution of each ICP compliance mechanisms *directly* in the same individual is not currently possible [5], we know that when neurons and glial cell cultures are subjected to high environmental pressure (analogous to elevated ICP), they rapidly adjust in volume through regulatory mechanisms [6–8], contradicting Monro and Kellie’s original assertions about the immutability of brain tissue [2]. We have demonstrated in vivo that large ischemic and hemorrhagic strokes can reduce brain parenchymal volume, with reduced cell volume and extracellular space in regions distal to stroke, such as striatal and hippocampal neurons (among other regions and cell types), termed “tissue compliance” [9, 10]. The magnitude of this effect suggests that tissue compliance is a significant factor in accommodating to high ICP after larger strokes (e.g., after ~ 75 µL striatal bleed in rat, CA1 soma size decreased by 42%, increased in cellular density by 34%) [9]. Vital translational considerations remain; for example, how does tissue compliance vary with age, across biological sex, or presence of comorbidities? This study begins answering these questions by examining the effect of age on ICP and tissue compliance.

Age is a key non-modifiable risk factor for stroke [11, 12], yet most stroke research uses young healthy male rodents, poorly representing clinical reality [13–15]. Although there is relatively little ICH data in aged animals, age has been associated with worse cell death [16, 17], edema [18], autophagy [19], and glial/immune dynamics [17–22], along with larger hematoma volumes and impaired hematoma resolution [17, 20], worse white matter damage [23, 24], and increased neurological deficits following ICH [18, 19]. This suggests that older animals should demonstrate worse ICP compliance, given increased vulnerability to severe strokes that cause widespread edema [18]. Conversely, younger patients are more likely to have elevated ICP values following an ICH versus older patients [25]. This could arise from selective patient attrition (higher mortality with age); alternatively, the numerous physiological and morphological changes within the aging brain (cerebral atrophy, impaired vasoreactivity/remodelling, impaired CSF outflow, and inconsistent blood pressure) affect ICP compliance [26–28]. These differences may provide more space and time for pressure to equalize following stroke, reducing the need for more costly “last resort” compliance mechanisms (e.g., tissue compliance and/or reductions in arterial blood flow), though this must be confirmed.

In humans, ICP usually averages 5–10 mmHg, but this varies with measurement approaches [1, 29]. In our hands, ICP typically averages ~ 4–5 mmHg in naïve adult rats [30–32], though others report wider ranging values [29]. Clinically, and in rodent models, ICP increases within hours following ICH, and can remain elevated for days [1]; a recent meta-analysis suggests that the pooled prevalence rate of intracranial hypertension (defined as ICP values > 20 mmHg) in ICH patients is 67%, with a pooled mortality rate of 50% [33]. Values exceeding ~ 40 mmHg have been reported in humans following primary ICH [34, 35], and in rats, we have shown that a moderately large ICH raises ICP by ~ 10 mmHg on average, with a peak ICP as high as 40–80 mmHg, depending on the model [32, 36]. The frequency of ICP values > 20 mmHg is associated with higher risk of death in humans and animal models of ICH [31, 32, 36, 37]. Currently, there are no studies measuring ICP in aged rats following ICH, especially given the challenge of collecting data without anesthetic confounds [1, 29, 36]. Clinical ICP monitoring is also not practiced routinely, as it is invasive [1, 38–40]. Accordingly, controversy exists regarding routine ICP measurement in ICH patients and impact on outcome, with some finding higher chances of infection and longer hospital stays [41], while others demonstrate reduced 6-month mortality [42]. Clinical ischemic stroke studies are similarly contradictory [43]. This is despite identification of various ICP parameters (e.g., time/frequency of ICP > 20 mmHg, ICP variability) as predictors of functional outcome and fatality following stroke and brain trauma [37, 40]. Given these controversies surrounding ICP monitoring and management in critical stroke patients, further translational data is needed to guide clinical practice. Therefore, this is the first paper to examine how aging affects ICP compliance following severe ICH.

First, to establish whether ICH mass effect induces tissue compliance in middle-aged rats, we assessed tissue compliance parameters for whole hemisphere and within various volume fractions (e.g., cortical thickness, ventricular volume, regional neuron density/volume) at 24 h after a severe striatal ICH. We used the collagenase model as it causes higher ICP compared to the whole blood model [10, 32]. Additionally, using telemetry to collect epidural pressure recordings [31] eliminates anesthetic confounds. Our hypothesis was that following ICH, aged rodents would engage in tissue compliance to a lesser degree compared to what we observed previously in young animals [9]. In experiment 2, ICP and ICP spiking events were recorded in middle-aged rats for 24 h following ICH, and brain water content (BWC) across striatum, hippocampus, and cerebellum was assessed; we hypothesized that ICP elevations and spiking behavior would be less than previously observed in young animals [36], while still displaying significant edema.

## Methods

### Animal Care

All experiments followed ARRIVE guidelines [44, 45] and Canadian Council on Animal Care Guidelines; procedures were approved by the University of Alberta Biological Sciences Animal Care and Use Committee (protocol #960). For experiment 1, 20 male Sprague–Dawley rats (50–75 g, 3–4 weeks old) were obtained from Charles River (Saint Constant, Quebec) and aged at University of Alberta until they reached 12 months of age. For experiment 2, 20 male Sprague–Dawley rats (9–11 months, retired breeders) were obtained and aged in our facility until 12 months old. Animals were group housed until 6–7 months of age when they were single-housed in a temperature and humidity-controlled environment, with ad libitum access to rat chow and water. The rooms were maintained at a 7:00–19:00 light cycle with manipulations performed during the light period.

### Experimental Design

Animals were assigned to experimental groups following aging using a random number generator (random.org) before manipulations, and all data was analyzed by experimenters blinded to group identity. Group sizes for experiment 1 were determined using an a priori power analysis to have > 80% power in detecting a 25% difference in neuronal volume and density, based on published data [9, 10]. Our sample sizes allowed for a < 0.1 average coefficient of error for each stereological endpoint. For experiment 2, minimum group sizes were calculated to have > 80% power in detecting a 1% change in BWC, which also permits 95% power to detect a 15% change in ICP between groups, based on our past work [36].

**Fig. 1:**
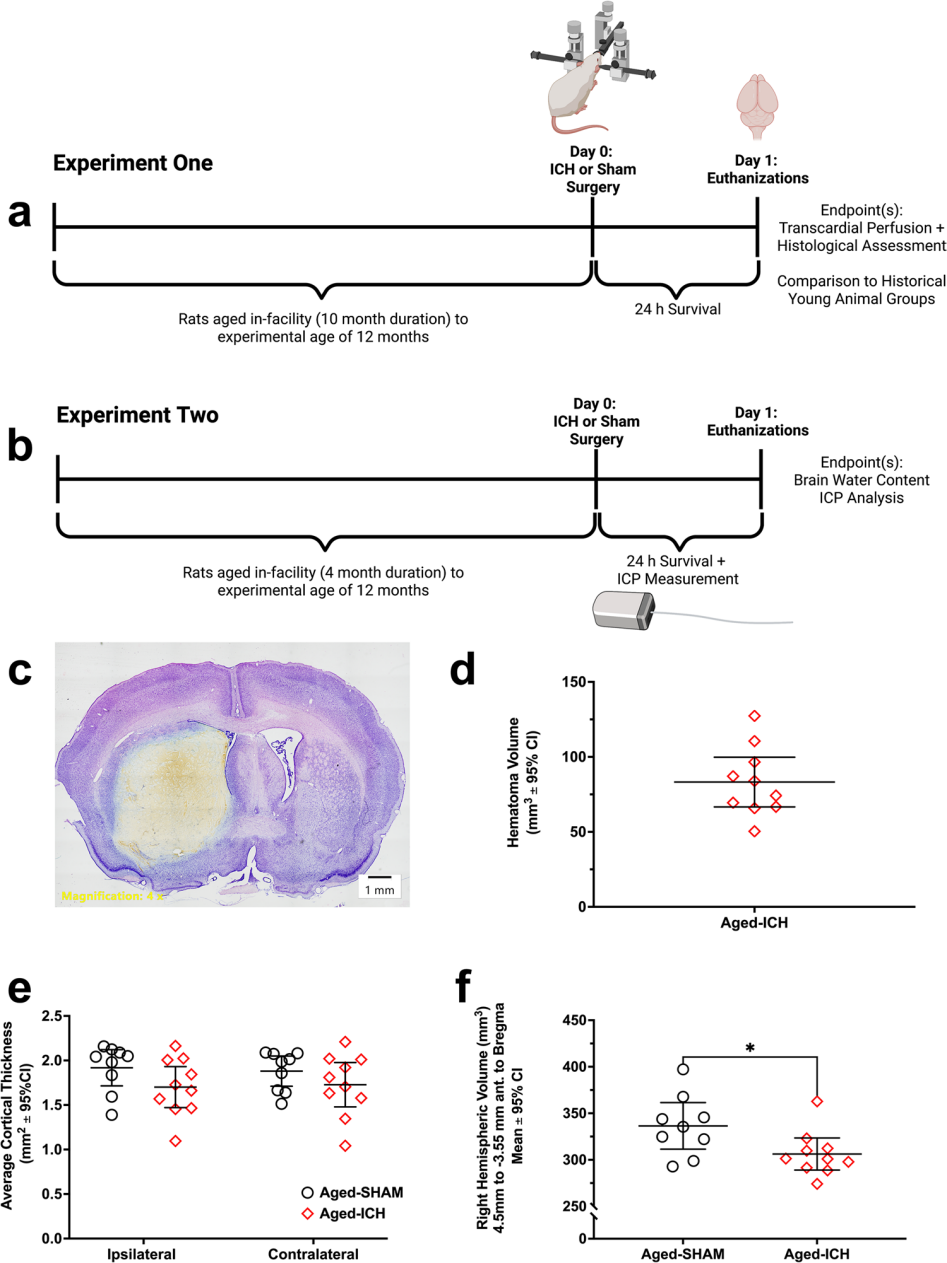
Experimental design and timelines (**a**, **b**); a representative cresyl violet section (**c**) demonstrates aged ICH hematoma volume at 24 h post-ICH in experiment 1 (**d**); note that portions of the hematoma often wash away during tissue processing, which is evident in fresh tissue. There were no significant differences in cortical thickness across hemispheres in aged ICH animals vs. aged SHAMs (**e**); however, aged ICH animals had significantly smaller right (contralateral) hemisphere parenchymal volume (total volume − ventricle volume), assessing from 4.5 to − 3.55 mm anterior to bregma (**f**). *p < 0.05 versus aged SHAMs

For experiment 1 (Fig. 1a), 20 aged rats were randomly assigned to receive either a collagenase-induced ICH (aged ICH, *n* = 10) or sham procedure (aged SHAMs, *n* = 10), and were euthanized 24 h post-surgery. Brain tissue was used to investigate cell volume and density bilaterally across CA1 and S1, and within contralateral striatum. The same tissue was used to assess hematoma volume, contralateral hemisphere, ventricles, and cortical thickness. Then, in a planned historical comparison, tissue from young animals (adult ICH, *n* = 7; adult SHAM, *n* = 9) was used to assess the impact of age on tissue compliance within brain volume fractions (contralateral hemisphere volume, ventricular volume, cortical thickness, CA1 neuron density/volume) 24 h post-surgery [9]. This minimized animal use, given that we have repeatedly demonstrated tissue compliance in young animals across multiple stroke models and severities [9, 10]. All tissues used in experiment 1 young animal comparisons were obtained from male Sprague–Dawley rats, weighing 350–400 g, ~ 2–3 months of age, from the same source (Charles River). Identical processing and surgical methods were used in both experiments [9]; data from adult ICH and adult SHAM groups were collected in August 2019, while work using aged ICH and aged SHAM groups in experiment 1 was carried out over October 2020–January 2021 as rats reached the desired age.

For experiment 2 (Fig. 1b), 20 aged rats were randomly assigned to receive either a collagenase-induced ICH and ICP probe implant (aged ICH, *n* = 10) or a sham operation and ICP probe implant (aged SHAM, *n* = 10). In all animals, ICP was recorded for 24 h after surgery until euthanasia. Brain tissue was taken for edema quantification using the wet-dry weight method [10]*.*

### Exclusion Criterion

For experiment 1, a priori exclusion criterion for stereological assessments was maintained to ensure optimal data accuracy (e.g., presence of bleed extension in region of interest, tissue/histological artifact), identical to the exclusion criterion used for the historical young animal dataset [9]. In experiment 2 only, a priori exclusion criterion for ICP data included probe failure, but not premature euthanasia due to stroke severity. We aimed to assess the full range of how aged animals comply with the added mass of a severe hemorrhage to better model the most critical human ICH patients; therefore, ICP data and brain tissue from prematurely euthanized animals were included in analyses to better represent these populations.

### Intracerebral Hemorrhage

To induce an ICH in the left striatum, rats were anesthetized with isoflurane (4% induction, 2.5% maintenance in 60% N_2_O, remainder O_2_) and secured in a stereotaxic frame. The procedure was performed as prior [9]. Briefly, a burr hole was drilled above the striatum (0.5 mm anterior and 3.5 mm left of bregma), and bacterial collagenase (type IV-S, Sigma, 3.0 μL of 0.6 U/μL in saline) was injected 6.5 mm below the skull surface over 5 min. Bupivacaine (0.1 mL, s.c.) was given as a post-operative analgesic under the incision site, and 0.9% saline (5 mL, s.c.) was given to prevent dehydration. In sham procedures, rats were kept under anesthetic for ~ 25 min, during which an incision was made but no burr hole was drilled; sham animals were given identical doses of bupivacaine and saline for recovery [9].

### Histological Processing

In experiment 1, rats were euthanized with sodium pentobarbital (100 mg/kg, i.p.) and transcardially perfused with 0.9% saline followed by 10% neutral buffered formalin (Fisher Scientific, Toronto, ON). Tissue was processed as described previously [9].

### Stereological Analysis

We assessed neuronal and astrocytic volume and density in areas CA1, S1, and striatum. All images were taken at 40 × magnification using an Olympus BX51 microscope with a DP74 CMOS camera (Olympus Corporation, Tokyo, Japan). Analyses were done on ImageJ (v.1.53p, NIH). A combined stereological approach was used to assess both cell packing density and volume, as published previously [9].

### Bleed Volume

In experiment 1, sections used for stereological analysis were also used to assess bleed volume. Images were taken at 1.25 × magnification using Olympus Cellsens Dimensions software (v.2.3) and analyzed using ImageJ. Sections were analyzed from approximately + 5.45 to − 6.00 mm bregma [9, 46].

### Cortical Thickness, Contralateral Hemisphere Volume, and Ventricular Volume

In experiment 1, average cortical thickness of each hemisphere was assessed in ImageJ by measuring distance from the dorsal point of the cingulum to the second cortical layer across five sections (ranging from approximately + 1.89 to − 2.0 from bregma) [9]. Whole contralateral hemisphere volume and lateral ventricular volume were determined by measuring area of each component within coronal sections ranging from 4.5 mm or 3.55, respectively, to − 3.55 mm anterior to bregma, also using ImageJ. Total volumes were calculated histologically across section intervals [46].

### Intracranial Pressure Recording and Analysis

In experiment 2, rats were implanted with PA-C10 pressure probes (Data Sciences Int., St. Paul, MN) to measure ICP [10, 30, 36]. This was following either collagenase infusion or sham procedure. Immediately after surgery, probes were turned on, collecting data at 30-s intervals for 24 h post-surgery. ICP data was processed and corrected for electronic or movement interference [10, 30, 36], which amounted to ~ 0.01% of raw data within each animal on average. ICP data was exported from ART, processed using Microsoft**®** Excel (v.16.61), blinded, and analyzed using Python script (Anaconda Python v.3.9; Spyder Package v.5.15). For detailed methods, refer to Online Resource 1.

Disproportionate increases in ICP (DIICP) and raised ICP (RICP) collectively characterize spiking and rising behavior in ICP following ICH [36, 40, 47, 48]. We defined DIICP events as a ≥ 10 mmHg increase in ICP for ≥ 3 min above the prior 60-min moving average (baseline reference), identical parameters to our past work [36]. We defined RICP events as an increase in ICP above ≥ 20 mmHg for ≥ 3 min when the baseline reference was also ≥ 20 mmHg, based on clinical definitions of intracranial hypertension [48]. This is the threshold at which risk of death in rodents increases [31, 32], and time spent above 20 mmHg is associated with ICH outcome and mortality [37, 40]. Other analyses included mean and peak ICP across minute-to-minute and 60-min epochs, and examining local ICP variability in the hour prior to flagged ICP events by examining rate of change (15-min slope) in order to assess intracranial compliance [40, 48, 49]. Any events that occurred in the first 120 min of the recording period were excluded, as pressure values are still normalizing post-surgery. Events flagged in aged ICH animals were compared to “aged SHAM equivalent” control values, which were determined by averaging 24 h of ICP data across aged SHAMs who did not experience any flagged ICP events.

### BWC

In experiment 2, at 24 h after surgery, rats were briefly anesthetized with isoflurane, decapitated, and brains were removed and assessed for BWC. Brains were blocked 2 mm anterior to 4 mm posterior from the stereotaxic location of the cranial burr hole drilled for epidural ICP measurements. Ipsilateral and contralateral striatum and hippocampus were extracted and weighed, along with cerebellum. After collecting tissue wet weights, samples were baked in the oven at 100 °C for 24 h, after which dry weights were determined. Total water content was expressed as [(wet weight − dry weight)/wet weight] $\times$ 100 [1].

### Statistical Analysis

All data was analyzed using GraphPad Prism (v.9.31, GraphPad Software Inc., La Jolla, CA). Statistical significance was set at *α* = 0.05, and all data are presented as mean ± 95% CIs; significant effect sizes are reported using mean difference ± 95% CIs, along with Cohen’s *d*. Assumptions of normality, heterogeneity, and sphericity were confirmed using the Shapiro–Wilk normality test, the *F* test, the Brown-Forsythe ANOVA test, and Spearman’s test for heteroscedasticity, respectively. All independent means were compared with unpaired Student’s *t* tests or one-way ANOVA followed up by Dunnett’s test. Parameters measured across both hemispheres in the same animal were compared using a two-way ANOVA with Sidak’s multiple comparison test; hemisphere (ipsilateral/contralateral) was the within-subject factor, and treatment group was the between-subject factor.

In experiment 2, ICP data was averaged into 60-min epochs over the entire 24 h recording period, and were compared across aged ICH vs. aged SHAMs using a two-way repeated measure ANOVA with Sidak’s multiple comparison test. Using the 60-min pre-event baseline period prior to each ICP event, slope (rate of change) was calculated across four 15-min epochs. Slopes were then compared across experimental groups over time again using a two-way repeated measure ANOVA with Sidak’s multiple comparison test. In relevant cases, the Greenhouse–Geisser correction was used. Lastly, Pearson’s *R* was used for correlating regional BWC levels and ICP parameters.

## Results

### Experimental Exclusions and Mortality

There was no mortality in experiment 1, but one aged SHAM rat was excluded based on a priori exclusion criteria for tissue quality. Three aged ICH rats died or were prematurely euthanized in experiment 2; however, according to a priori criteria, data from these animals were included in all ICP analyses. Of these 3 mortalities, two animals were found dead in their home cages in the morning, while one was prematurely euthanized at ~ 6 h post-ICH; visible signs of brain herniation at the foramen and papilledema, paired with large pre-death ICP spikes implicated high ICP as a cause of death. Brain tissues from the prematurely euthanized animal were included in edema measurements to minimize loss of tissue from costly aged animals, as a priori exclusion criteria for edema quantification dictated.

### Experiment 1

#### Histological Bleed Volume

As expected, the hematoma was largely confined to left striatum, and it was a large stroke (83.22 mm^3^). Aged SHAMs had no injury (Fig. 1c, d).

#### Cortical Thickness, Whole Right Hemisphere Volume, and Ventricular Volume

Compared to aged SHAMs, aged ICH animals did not have significant differences in ventricular volume (*p* = 0.6098; Fig. 4c). Though aged ICH animals had relatively thinner cortices at 24 h post-stroke both ipsilaterally and contralaterally compared to aged SHAMs (by a mean difference of 10% ± a 95% CI of 11%), this was only a trend (*p* = 0.0622). There were no significant differences between hemispheres, and no interaction (both *p* $\ge$ 0.2232; Fig. 1e). The average volume of the right (contralateral) hemisphere interval was significantly lower in aged ICH animals compared to aged SHAMs (10% ± 9%; *p* ≤ 0.05; Cohen’s *d* = 1.05; Fig. 1f); this measurement solely reflects total parenchymal volume, as ventricle volume was subtracted. Therefore, aged ICH animals experienced a significant reduction in total parenchymal volume within the right hemisphere, 24 h post-stroke.

#### Neuron Cell Volume and Density (CA1, S1, Striatum)

**Fig. 2:**
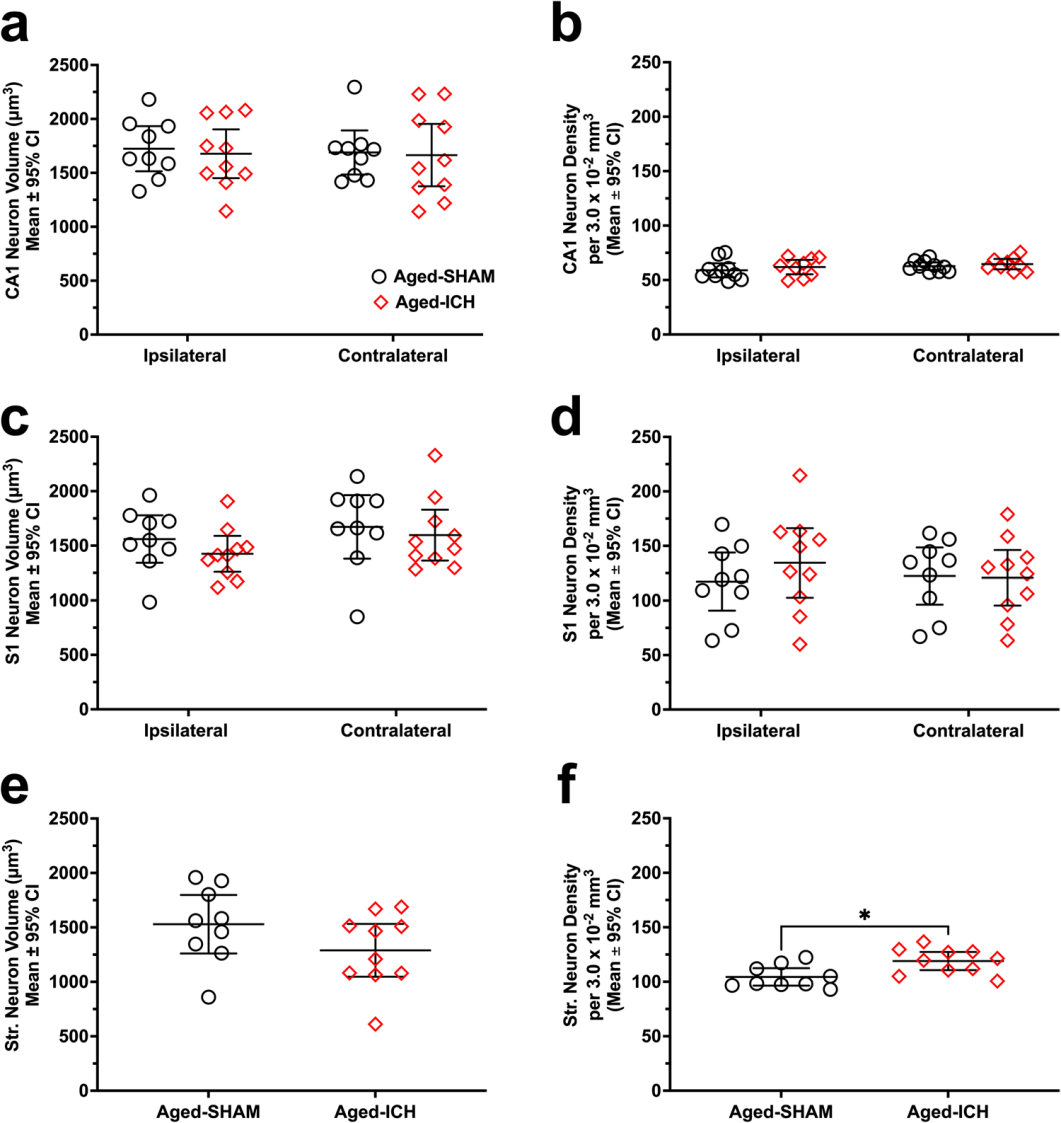
In experiment 1, neuron soma volume (**a**, **c**, **e**) and density (**b**, **d**, **f**) were assessed in aged rodents after either ICH or sham procedure. There were no significant differences in cell volume or density when assessing bilaterally in the hippocampal CA1 layer (**a**, **b**) and cortical area S1 (**c**, **d**). Assessment of the striatum was limited to the contralateral side of the brain, as the ipsilateral striatum was largely destroyed by the hematoma in ICH animals; there were no significant differences in neuron cell volume here (**e**), but there was a significant increase in aged ICH striatal cell density vs. aged SHAMs (**f**). *p < 0.05 versus aged SHAMs

In aged ICHs, average CA1 neuron volume and density were only 3% and 4% lower bilaterally vs. aged SHAMs, respectively, and did not differ significantly by hemisphere or treatment, with no interactions (*p* $\ge$ 0.1845; Fig. 2a). Although aged ICH neuron volume and density in S1 were also lower and higher, respectively, compared to aged SHAMs, these differences were not significant by treatment or by hemisphere, with no interactions (*p* $\ge$ 0.1666; Fig. 2c, d). Average striatal neuron volume in the contralateral hemisphere was 16% lower compared to aged SHAMs, but did not differ significantly at 24 h (*p* = 0.1484; Fig. 2e). However, contralateral striatal neuron density was significantly higher compared to aged SHAMs, by 12% ± 9% (*p* ≤ 0.05; Cohen’s *d* = 1.32; Fig. 2f)*.* Therefore, there is some evidence for tissue compliance in the aged ICH group, although in many comparisons, the findings were statistically non-significant, owing to a higher degree of variability reflective of age-related differences in cellular compliance.

#### Astrocyte Volume and Density (CA1, S1, Striatum)

**Fig. 3:**
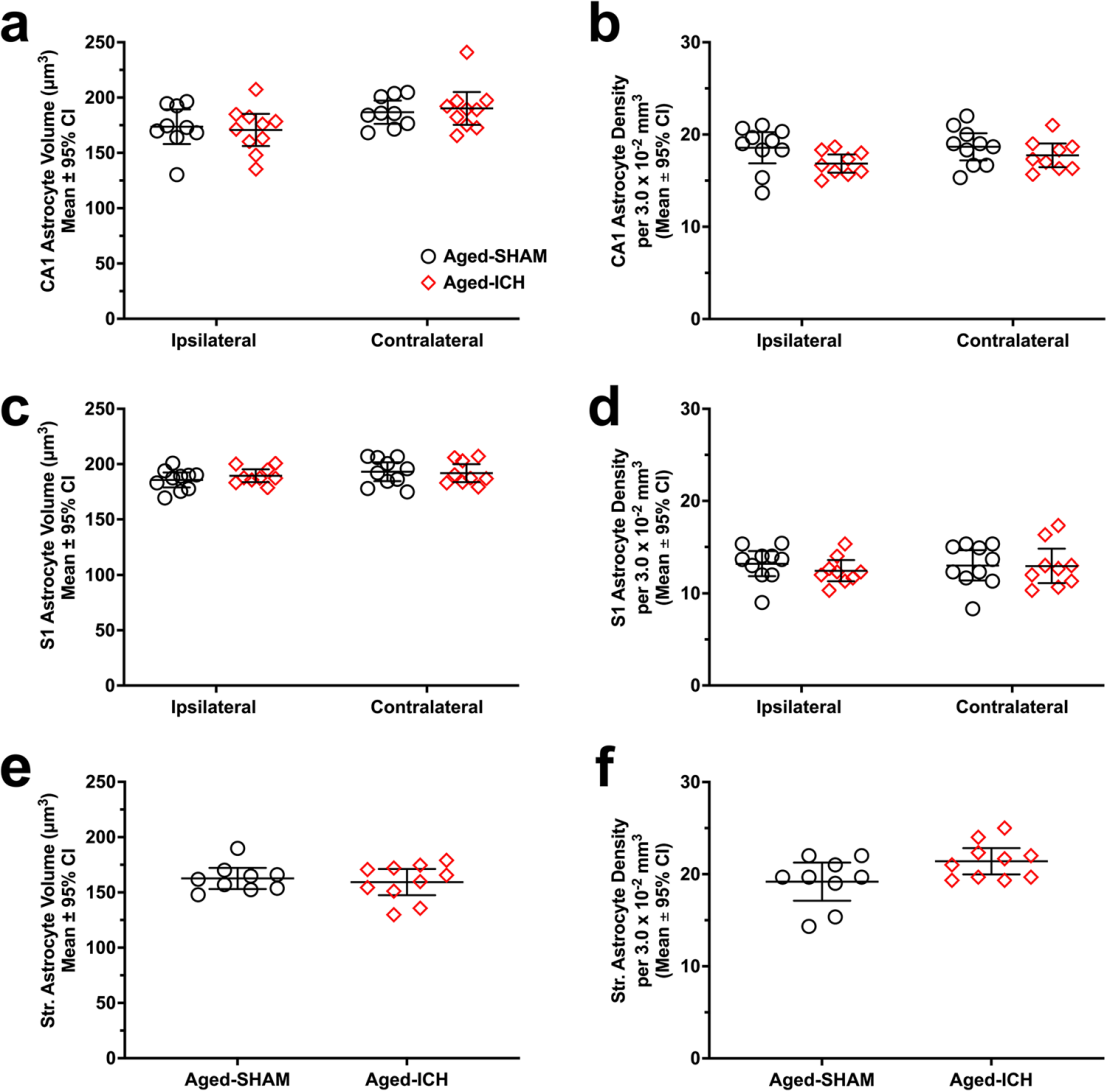
In experiment 1, astrocyte soma volume (**a**, **c**, **e**) and density (**b**, **d**, **f**) were assessed in aged rodents after a striatal ICH or sham procedure. Regions analyzed bilaterally included the CA1 zone (**a**, **b**) and area S1 (**c**, **d**), while striatum was analyzed only in the right hemisphere (**e**, **f**). Astrocyte volume in CA1 was significantly different across hemispheres, but not across experimental groups, a baseline effect observed prior in other aging experiments (**a**). Astrocyte density in CA1 was significantly higher in aged ICH animals vs. aged SHAMs (**b**). There were no significant differences across experimental groups or hemispheres in area S1 and striatum; however, striatal astrocyte density trended on being significantly higher in aged ICH animals, similar to what was observed in neuronal morphological data

Across experimental groups, CA1 astrocyte volume differed significantly by hemisphere (*p* ≤ 0.01; Cohen’s *d* = 0.86), with an ipsilateral (left) hemisphere CA1 astrocyte volume 9% ± 7% lower than the contralateral (right) hemisphere; however, there was no significant effect of treatment group, and no interaction (both *p* $\ge$ 0.6109; Fig. 3a). Conversely, CA1 astrocyte density differed significantly by treatment group (*p* ≤ 0.05; Cohen’s *d* = 0.51), where the aged ICH group had a 7% ± 6% higher CA1 astrocyte density vs. aged SHAMs (Fig. 3b); there was no significant differences across hemisphere, with no interaction (both *p* $\ge$ 0.4302). In area S1, astrocyte volume and density did not differ significantly across hemisphere or treatment group, with no interactions (all* p* $\ge$ 0.1422; Fig. 3c, d). Similarly, in contralateral striatum, astrocyte volume did not differ significantly across groups (*p* = 0.6327; Fig. 3e); however, there was a trend towards higher astrocyte density in this region within aged ICHs (7% ± 7% vs. aged SHAMs; *p* = 0.0561; Fig. 3f), similar to what was observed in other regions and cell types.

#### Planned Historical Comparison to Young Adult Animals

**Fig. 4:**
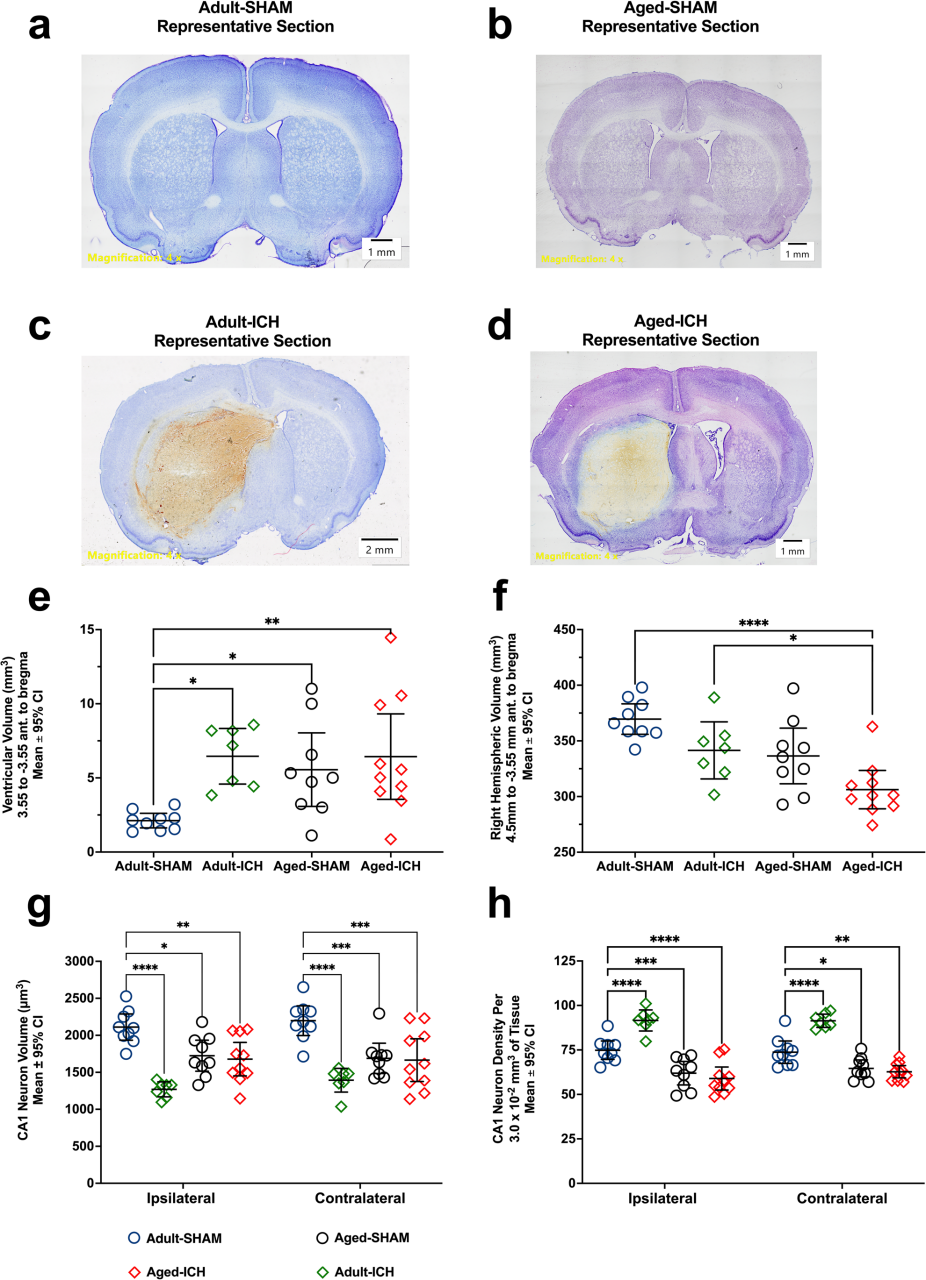
Representative brain sections from adult SHAM and aged SHAM animals (a, b), as well as adult ICH and aged ICH animals (c, d) demonstrating an increased ventricular volume (e) that is accompanied by a reduction in total right hemispheric parenchymal volume (d) as this rodent strain ages. Additionally, CA1 neuron volume (f) and density (g) in aged SHAM and aged ICH animals were significantly lower compared to adult SHAMs, nearing values observed in adult ICH animals. Therefore, following an ICH in an aged animal, it is possible that a lesser degree of tissue compliance is needed to accommodate the mass of a hematoma compared to their younger counterparts, as there is likely less resistance to CSF outflow through the ventricular system (c, d). *p < 0.05, ** p < 0.01, ***p < 0.001, ****p < 0.0001 versus adult SHAMs

To establish how tissue compliance changes with age, we compared experiment 1 aged animal data to our past data in young adult animals, who were euthanized at 24 h post-ICH using identical methods to the present study (adult ICH/SHAM vs. aged ICH/SHAM; Fig. 4a, b) [9]. We found significant evidence of age-related atrophy in middle-aged animals. Compared to adult SHAMs, the aged ICH and aged SHAM groups had larger ventricular volumes (67% ± 51% and 62% ± 61%, respectively; both *p* ≤ 0.05; Cohen’s *d* $\ge$ 1.47; Fig. 4c), along with a smaller overall contralateral hemisphere volume (17% ± 9% and 9% ± 5%, respectively; both *p* ≤ 0.05; Cohen’s *d* $\ge$ 1.26*;* Fig. 4d). Interestingly, ventricle volume in adult ICH animals was also significantly enlarged (67% ± 55% vs. adult SHAMs; *p* ≤ 0.05; Cohen’s *d* = 2.88; Fig. 4c); at this acute survival time, this could reflect obstruction of CSF drainage following ICH, or ventricular mid-line shift/displacement, but this remains to be seen. The age-related ventriculomegaly and cerebral atrophy was not accompanied by significant cortical thinning; though cortical thickness differed significantly by experimental group (*p* ≤ 0.01), further analysis demonstrated that only adult ICH animals had significantly lower values vs. adult SHAMs (14% ± 12%; *p* = 0.0372; Cohen’s *d* = 2.38; Online Resource 1; Fig. 7). This could be explained by regional variations in cerebral atrophy rates occurring across both animals and humans, as we assessed cortical thickness through a limited interval of brain sections along the rostral-caudal axis.

When examining how CA1 neuron volume and density differ with age, we found that CA1 neuron volume was significantly smaller in both aged ICH and aged SHAMs compared to adult SHAMs (21% ± 11% and 22% ± 11%, respectively; both *p* ≤ 0.0001; Cohen’s *d* $\ge$ 1.57), similar to that of adult ICH animals 24 h post-ICH, which occurs due to tissue compliance (38% ± 12%; *p* ≤ 0.0001; Cohen’s *d* = 4.10). However, unlike adult ICH animals (who display a 19% ± 7% increase in CA1 cell density vs. adult SHAMs; *p* ≤ 0.0001; Cohen’s *d* = 2.49), aged ICH animals had a 18% ± 7% lower cell density vs. adult SHAMs, while aged SHAMs displayed a 15% ± 8% lower cell density (both *p* ≤ 0.0001; Cohen’s *d* $\ge$ 1.40). This could indicate ongoing age-related atrophy and/or vulnerability to injury. Therefore, age plays an important role in determining the timing and occurrence of tissue compliance dynamics in response to severe ICH.

### Experiment 2

#### Intracranial Pressure

**Fig. 5:**
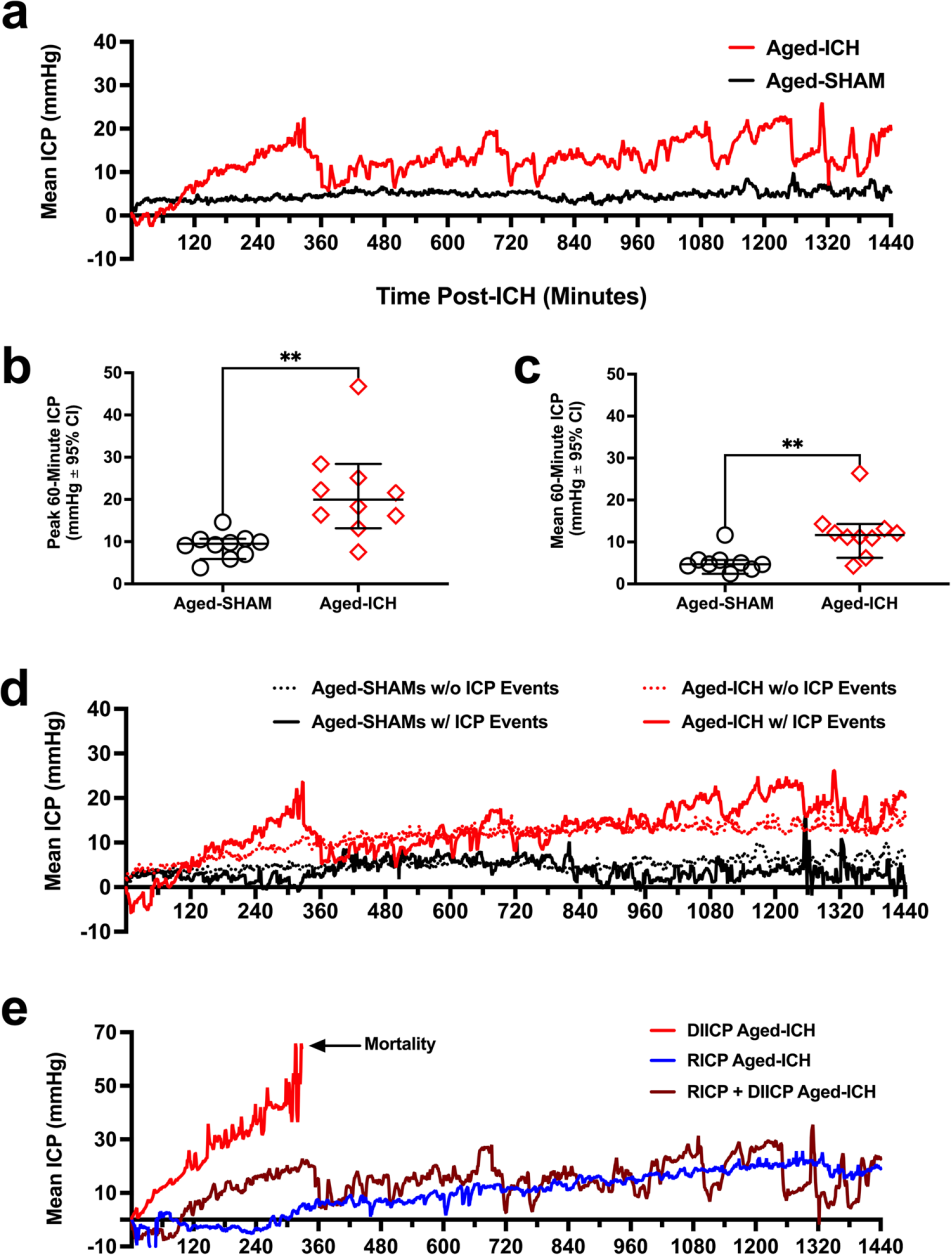
In experiment 2, ICP recordings were taken for 24 h in aged ICH animals and aged SHAMs (*n* = 10 per group); average 60-min ICP increased significantly in the aged ICH group at 12–24 h post-ICH vs. aged SHAMs (**a**). Aged ICH animals had significantly higher mean and peak ICP over 60-min epochs compared to aged SHAMs across the entire 24-h period (**b**, **c**), indicating acute ICH mass effect. Aged ICH and aged SHAM groups were further divided into those that spontaneously experienced ICP events (5 aged ICH animals, 2 aged SHAMs), and those that did not (5 aged ICH animals, 8 aged SHAMs; **d**). These ICP events are associated with adverse outcomes both in rodents and humans, displaying uncharacteristic deviations away from baseline, such as DIICP and RICP events (defined in main body of text). The aged ICH animals that experienced both RICP and DIICP events (*n* = 2), RICP events only (*n* = 2), or DIICP events only (*n* = 1; spontaneous mortality) are shown in panel **e**; such events are indicative of poor intracranial compliance. **p < 0.01 versus aged SHAMs

In experiment 2, when comparing 24 h of ICP data averaged in 60-min epochs in aged ICH animals vs. aged SHAMs, time, treatment group, and the interaction between these main factors were all significant (*p* ≤ 0.0026; Fig. 5a). ICP was significantly elevated from 11 to 24 h post-surgery in aged ICH animals compared to aged SHAMs (mean difference of 8.93 mmHg ± 7.04 mmHg, or 60% ± 47% averaged over 11–24 h; Cohen’s *d* $\ge$ 2.50; all *p* ≤ 0.0371; Fig. 5a). Additionally, ICP reached significantly higher mean and peak magnitudes in aged ICH animals averaged across 60-min epochs (mean difference of 12.51 mmHg ± 7.79 mmHg and 7.43 ± 4.36, respectively, or 60% ± 36%; all *p* ≤ 0.01 vs. aged SHAMs; Cohen’s *d* $\ge$ 1.59; Fig. 5b, c). These findings reflect compromised compliance after ICH.

#### DIICP + RICP Events

**Fig. 6:**
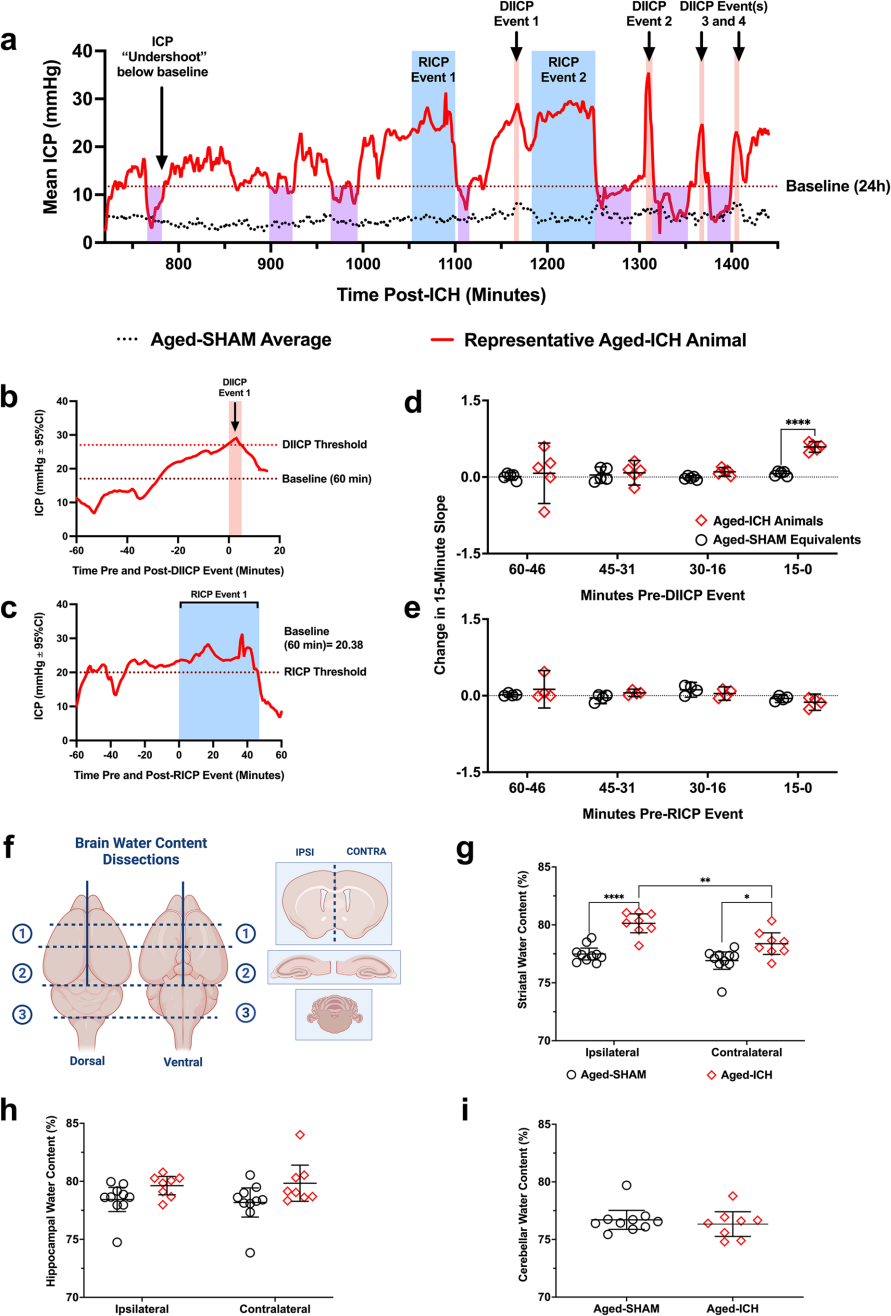
Representative ICP trace in an aged ICH animal displaying all ICP events flagged over the 24 h recording period in experiment 2 (**a**); examples of the baseline reference period are shown (average ICP over the preceding 60 min prior to the DIICP/RICP event), in panels **b** and **c**. In aged ICH animals, the 60-min baseline reference period prior to each event was split into 15-min epochs, and change in mean ICP slope was calculated for each epoch, comparing to equivalent periods averaged across aged SHAMs (**d**); a significant increase in pre-event slope occurred over the 15–0 min epoch immediately prior to DIICP events across aged ICH events vs. time-matched aged SHAMs, indicating worse control of cerebral compliance reserves (**e**). Following ICP recordings, animals were euthanized, and the striatum and hippocampi were dissected out bilaterally for BWC, along with the cerebellum (**f**). In the striatum of aged ICH animals, BWC was significantly elevated across both hemispheres vs. aged SHAMs, with significantly higher BWC in the ipsilateral (left) vs. contralateral (right) hemisphere (**g**). Aged ICH animals had significantly higher brain water content bilaterally in the hippocampus vs. aged SHAMs, also indicating widespread edema (main effect of group; *p* ≤ 0.05; **h**). As expected, brain water content in the cerebellum was not elevated in aged ICH animals compared to controls (**i**). *p < 0.05, ** p < 0.01, ***p < 0.001 vs. aged SHAMs

In the first 120 min post-surgery, two aged ICH animals had DIICP events, which were excluded, for reasons stated above. Following the first 120 min, 50% of aged ICH animals had an ICP event over the 24-h period: of the aged ICH animals that experienced ICP events, 40% experienced both DIICP and RICP events, 40% experienced only RICP events, and 20% experienced only DIICP events (Fig. 5d, e; S.M. Table 1). Two aged SHAMs also experienced a brief 4-min DIICP event (Fig. 5d; S.M. Table 1); however, none of the aged SHAMs experienced RICP events. The average first occurrence for DIICP events in aged ICH animals was at 12.75 h post-ICH, while for RICP events, it was 19.5 h post-ICH. The average event duration for DIICP events was 4.25 min in aged ICH animals, with 8 different events flagged; this was versus 4 min in aged SHAMs, across 2 events. The average event duration for RICP events was 30.38 min in aged ICH animals, with 16 different events flagged. A representative ICP trace from an aged ICH animal who experienced both DIICP and RICP events is shown in Fig. 6a. For more information, refer to Table 1, Online Resource 1.

#### Local ICP Variability Within Pre-event Baseline Reference Period(s)

ICP variability is a better predictor of long-term outcome in patients over mean ICP and identifies those with sub-optimal ICP compliance or poor cerebral autoregulation [50]. The slope of mean ICP indicates greater changes in ICP over time, identifying those whose intracranial compliance reserves are failing [51], and predicting upcoming ICP events [52]. To confirm whether this occurs in our model, data from each pre-event baseline period (e.g., the 60 min prior to each DIICP or RICP event as identified by our code; Fig. 6b, c) were split into four 15-min epochs (60–46 min, 45–31 min, 30–16 min, and 15–0 min pre-event). These pre-event slopes were averaged within each animal, and aged ICH animals who experienced DIICP or RICP events were compared to aged SHAM equivalent time-matched mean data; there was a significant effect of experimental group and time, as well as a time by group interaction when comparing pre-event DIICP slopes (all *p* ≤ 0.05; Fig. 6d). Further comparisons demonstrated that there was a significant increase in pre-event slope over the last 15-min epoch prior to the DIICP event vs. time-matched aged SHAM events (90% ± 25% change in ICP over time; *p* ≤ 0.0001; Cohen’s *d* = 7.72; Fig. 6d), but not over the first three 15-min epochs prior (all *p* ≥ 0.7082), mirroring human patients. The pre-event slopes prior to RICP events did not differ significantly across group, time, or subject, with no interactions (all *p* ≥ 0.0619; Fig. 6e). We acknowledge that these variability analyses are underpowered and exploratory.

Therefore, ICH in aged animals resulted in spiking and rising ICP events (Fig. 6a–c), and as seen in humans [48, 51, 52], a non-linear increase in ICP slope occurs in the 15 min before a spiking (DIICP) event (Fig. 6d). Those ICH animals that experienced only early DIICP events had worse outcomes (spontaneous death), while those with only RICP events generally had less ICP variability compared to those who experienced both (Figs. 5e and 6d, e), as confirmed by local variability analysis (Fig. 6d, e). These differences likely reflect individual variation in intracranial compliance reserves, and the capacity to accommodate mass effect.

#### BWC

Brain water content (Fig. 6f) varied significantly by hemisphere, region, and experimental group (*p* ≤ 0.01). Aged ICH animals had higher striatal water content in both hemispheres (3% ± 2% ipsilaterally, 2% ± 1.8% contralaterally; *p* ≤ 0.01 versus aged SHAMs; Cohen’s *d* $\ge$ 1.36; Fig. 6g). In the hippocampus (Fig. 6h), BWC percentage was significantly affected by treatment group (2% ± 1%; *p* ≤ 0.05; Cohen’s *d* = 0.94), but not hemisphere, with no interaction (both *p* ≥ 0.6568). Cerebellar BWC did not differ significantly between groups (*p* = 0.5312; Fig. 6i), as expected. Upon dissection, there were no signs of contralateral bleed extension; therefore, the effects observed in the contralateral hemisphere are likely in response to the ICH and tissue compliance, rather than the result of serum extrusion from the hematoma. Across all regions assessed, there were no significant correlations between edema and average or peak 60-min ICP (all *R*^2^ ≤ 0.13, *p* ≥ 0.3892). Therefore, striatal ICH resulted in significant edema that did not correlate with peak or average ICP.

## Discussion

Using the collagenase model, we produced a severe ICH with substantial brain swelling in middle-aged rats. This significantly raised average and peak ICP, and caused RICP and DIICP events, all indicating poor intracranial compliance. Established mechanisms of compliance include reductions in CSF and intravascular blood volumes. More recently, tissue compliance was demonstrated in adult rats after severe strokes [9, 10]. Similarly, in middle-aged rats, we observed an ~ 9% reduction in the contralateral hemisphere volume. At the cellular level, however, there were fewer indicators of tissue compliance in aged rats (e.g., significantly higher density of contralateral striatal neurons), unlike the much greater and widespread volumetric and density changes seen in young adult rats with comparable bleeds [9]. While further work is needed to understand age-related changes in tissue compliance, our data suggest that age-related brain atrophy plays a role (e.g., cell volume decline, ventricular enlargement, and hemisphere shrinkage). We observed that at minimum, aging resulted in 1% higher ventricular volumes and 5% parenchymal volume reduction over ~ 50% of the contralateral hemisphere (measured over section intervals). Even if this atrophy is a regionally specific effect, it still represents a possible ~ 20 μL of intracranial space alone (assuming ~ 1500-mm^3^ brain volume in aged rodents). Such savings are meaningful, given that this is 24% of the mean hematoma volume observed in experiment 1. These findings are important steps towards discerning the translational relevance of aged rats in ICH research, specifically regarding perturbation of intracranial compliance following large bleeds.

Many ICH animal studies measure BWC to gauge edema therapeutics [15]. Most commonly, edema is inferred from water content measures of tissue dissected from the injured hemisphere, and compared to control animals and unaffected brain regions. Despite its simplicity, these data are confounded, as elevations in water content arise not only from vasogenic edema but also from serum extrusion, and readings vary by amount of undamaged tissue within the sample. Additionally, our work in young rats [10, 53] and here in middle-aged rats show that BWC poorly predicts common ICP endpoints within ICH groups. Undoubtedly, this is due to the complex interplay of factors causing mass effect and mechanisms of intracranial compliance. More broadly, edema-ICP relationships vary over time; they are influenced by comorbidities (e.g., advancing age and health conditions [54]), therapeutics (e.g., hypothermia [53, 55, 56]), or methodological confounds (e.g., anesthesia during ICP monitoring [57–59]). Thus, we urge caution using BWC as a primary endpoint; instead, we recommend additional collection of continuous ICP recordings without anesthetic confounds, as cerebrovascular resistance, cerebral blood flow, and mean arterial pressure are affected during anesthetic exposure, directly impacting ICP [57–60].

**Fig. 7:**
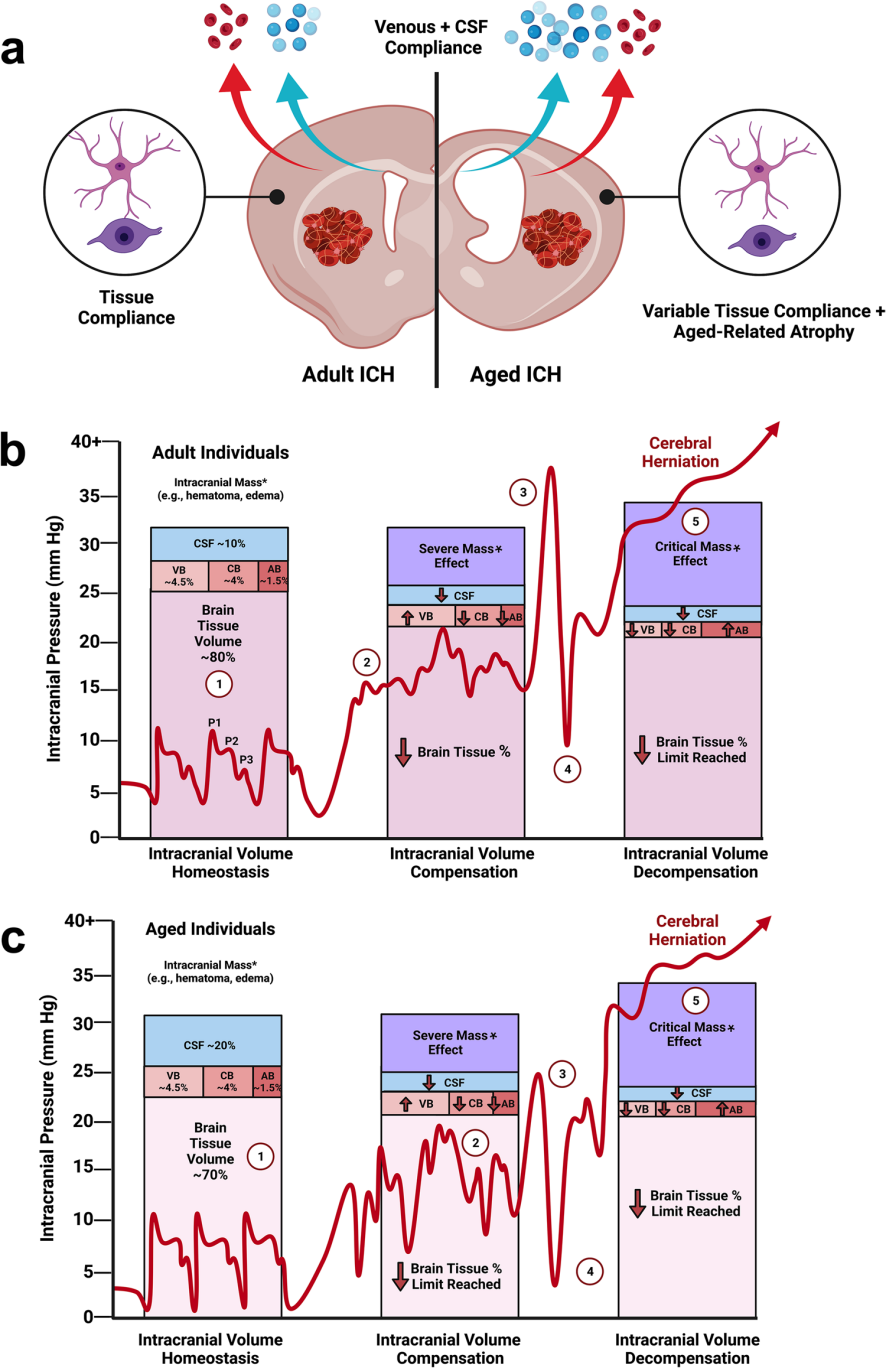
A summary of theorized tissue compliance dynamics that take place with age (**a**); increased ventricular size and decreased parenchymal volume that occur in the brain with age play a role in ICP compliance, changing the dynamics of accommodating ICH-associated mass effect (**b**, **c**). This theorized difference in ICP dynamics in young adults vs. aged individuals in response to cranial mass effect is illustrated by (1) brain at intracranial volume homeostasis. Typical “P1,” “P2,” and P3,” idealized ICP waveforms are shown (red line), reflecting intracranial compliance and respiratory rhythms; (2) brain at intracranial volume compensation. Added mass results in redirection of blood and CSF from and within the cranium, and a reduction in brain tissue volume as compensatory measures, deviating from typical ICP waveform patterns; (3–4) balancing the fluctuation in CSF and venous/capillary blood volume can cause dramatic spikes (3) and undershoots (4) in ICP away from baseline as mass effect grows more severe, reflecting periods of hyper and hypo cerebrovascular perfusion. Lastly, (5) as mass effect reaches a critical point and compliance mechanisms are exhausted, ICP begins to increase and loses its distinctive waveform as it climbs (e.g., intracranial volume decompensation). This is associated with a high risk of cerebellar herniation and mortality. With age, cerebrovascular reactivity, CSF redirection, and potentially tissue, compliance does not occur as rapidly or as effectively, causing refractory dynamic instability in ICP away from baseline and increasing risk of poor outcome or mortality. Figure created using BioRender.com (Toronto, Ontario)

Generally, our ICP findings and mortality rate in aged rats concur with our earlier work in young adult rats with roughly comparable bleeds in the collagenase model [30, 32, 53], but ICP levels are higher than that observed in the autologous whole blood model [10]. Our past work showed that the risk of mortality is highest in those with average ICP values exceeding 20 mmHg. Interestingly, the rat who experienced only DIICP events and the highest elevation in both mean and peak ICP was also a premature mortality in experiment 2 — all suggestive of a failure of compliance reserves. Those who experienced both DIICP and RICP events displayed greater ICP variability and higher mean ICP over the recording period, also reflective of ongoing depletion of compliance reserves. Animals who experienced only RICP events had less ICP variability and lower mean and peak ICP, possibly due to better maintenance of compliance reserves and cerebral autoregulation. Finally, others exhibit no events after ICH, potentially attributable to smaller bleed sizes, atrophy, and/or better maintenance of cellular volume control and other compliance mechanisms, though these hypotheses remain to be tested. A few aged SHAMs exhibited brief DIICP events, but these were brief and of a smaller magnitude compared to aged ICH DIICP events — perhaps aging in itself worsens control of cerebral compliance reserves and ICP homeostasis, along with known effects on cerebral autoregulation. Altogether, these findings indicate that our model fits with clinical ICP data in numerous ways (elevated peak and mean ICP, occurrence of both RICP and DIICP events, and significant change in ICP slope prior to DIICP events), albeit with some species-specific differences (e.g., lower baseline ICP, mass effect timing, different respiratory and cardiac rhythms and volumes) (Fig. 7) [1].

ICP spiking events (DIICPs and RICPs) collectively indicate poor intracranial compliance, and are commonly observed following ICH [36], among other disorders [48, 52, 61–63]. Specifically, DIICP events resemble “Lundberg A” slow-wave ICP waveforms (e.g., occurring over scale of minutes). Lundberg A slow waves are characterized by pathological elevations and plateaus in ICP lasting 3–10 min, often followed by a dramatic drop in ICP, often lower than baseline (e.g., “ICP undershoot”) [64]. This reflects ongoing cerebral autoregulation and activation of ICP compliance mechanisms. In scenarios where cerebral autoregulation is compromised, such as with age and after stroke [65, 66], deviations from baseline ICP can result in periods of cerebral hyperperfusion and hypoperfusion. A temporal mismatch between pressure mechanosensation, pressure-flow relationships, and cerebrovascular reactivity then contributes to secondary injury, cyclically exacerbating ICP dysfunction [67]. Therefore, growing amplitude and duration of Lundberg A waves indicates exhausted ICP compliance reserves. Maximizing “safe” redirection of blood, CSF, and active brain tissue volume regulation critically warn of an impending risk of secondary ischemic and mechanical injury (e.g., brainstem herniation) [9, 64, 68]. Further work is needed on these issues in ICH models, although this is complicated by the inability to concurrently measure multiple parameters in rats (e.g., state of each compliance mechanism, ICP, and cerebral blood flow).

Tissue compliance peaked 24 h after ICH in young rats and was evident in hippocampus, cortex, and striatum [9]. In both young and aged rats, we estimate that tissue compliance can accommodate an additional ~ 130 µL (95% CI ranges ~ 20–250 µL) if extrapolating the effect we observed in contralateral hemisphere across the brain. However, unlike young rats, fewer differences in neuron or astrocyte cell volume and density were observed in aging rodents — leaving the possibility that contralateral hemispheric shrinkage resulted from cumulative but smaller changes across regions, cell types, and perhaps other compartments that were not evaluated (e.g., neuropil). Further experimentation is needed to uncover mechanisms of tissue compliance and age-related influences, including larger CSF fraction in older rats owing to brain atrophy — this may allow a greater role of CSF outflow in intracranial compliance. This is complicated by the fact that CSF production rates and outflow resistance change with age, and do not necessarily relate linearly to changing lateral ventricle size [69]. The age-related cerebral atrophy is well documented in animals [70] and humans [71], where gray and white matter volumes steadily decrease after middle age, with increased ventricular volume. Both aging humans and animals experience cerebral atrophy, which occur at varying rates across regions. The relative atrophy seen in our rats roughly fits with the level of atrophy observed in 50–60-year-old humans [71].

These and other age-related changes heavily depend on cumulative lifestyle factors, such as hypertension, smoking, hyperlipidemia, and hyperglycemia [72–74], so it is not surprising that individuals experience varying rates of brain atrophy with age. Regardless, reasons for older individuals having small neurons and astrocytes compared to younger counterparts are still not well understood. Atrophy occurs at 5–10% per decade after 40, accelerating after age 70; regardless, cell loss is estimated at only 2–4% per decade [75]. While neuron density decreases slightly with age, macroscopic brain volume evaluations demonstrate appreciable atrophy [71], attributable to loss of cell volume and neuropil. Given the important role of tissue compliance, these age-related changes to cerebral morphology likely determine an individual’s ability to accommodate life-threatening cerebral masses. This could make the difference between a recoverable ICH and death when considered alongside other compliance factors and health of cerebral autoregulatory mechanisms. Mapping how these processes relate between humans and animal models of ICH will aid the design and delivery of future therapeutics, but vitally, the impact of age on parameters that determine and affect ICP must be better explored, such as to improve chances of translational success.

Our study has limitations. Primarily, the inability to concurrently measure the contribution of all ICP compliance mechanisms limits determination of the role of each one. The use of aged animals, along with telemetric ICP recordings, each with its inherent difficulties and costs, limited our sample size. Comparison of ICP events within the subset of aged ICH animals that experienced them somewhat reduces generalizability of conclusions for these analyses; nonetheless, we observed a high degree of consistency with clinical data [35, 37, 47–52]. Despite justification, comparison of aged animals to previous work (historical control) is also potentially problematic. Our study did not address underlying mechanisms (e.g., osmotic volume regulation) of tissue compliance, along with sub-cellular injury, and how these mechanisms vary with age and comorbidities, an area we are investigating. Finally, we did not assess how potential therapies impact ICP and tissue compliance responses across ages, a future research aim. Answering these questions will ultimately help develop new treatments for severe strokes, and milder insults that also affect ICP. Our data demonstrates that differences in cell volume, hemisphere volume, ventricular volume, and cortical thickness occur with age, likely affecting the ICP response to stroke. Therefore, intracranial compliance capacity becomes more heterogenous with age, arising from the highly individualized interplay between atrophy, tissue compliance, CSF outflow, and numerous other factors. These age-related differences may dictate the course and dynamics of ICP following an ICH, parameters directly related to the extent of “secondary” stroke injury and increased risk of mortality.

### Supplementary Information

Below is the link to the electronic supplementary material.

Supplementary file1 (DOCX 2327 kb)

Supplementary file2 (XLSX 755 kb)

Supplementary file3 (XLSX 2042 kb)

Supplementary file4 (PDF 123 kb)

## Data Availability

All data and code are available as supplementary material.
