# Supplementary material for: Intracranial Pressure Dysfunction Following Severe Intracerebral Hemorrhage in Middle-Aged Rats
Source: Transl Stroke Res. 2022 Nov 11;14(6):970–86. doi: 10.1007/s12975-022-01102-8 (PMC10640482; doi:10.1007/s12975-022-01102-8)
Supplement: Supplementary file 1 — Supplementary file1 (DOCX 2327 kb) [file 12975_2022_1102_MOESM1_ESM.docx]

**Supplemental Material 1**

**Manuscript Title: Intracranial Pressure Dysfunction Following Severe Intracerebral Hemorrhage in Middle-Aged Rats**

Anna C. J. Kalisvaart^1^, Ashley H. Abrahart^1^, Alyvia T. Coney^1^, Sherry Gu^2^, Frederick Colbourne.^1,2^

Author Information:

1. University of Alberta, Department of Psychology; Edmonton, Alberta, Canada.

2. University of Alberta, Neuroscience and Mental Health Institute; Edmonton, Alberta, Canada.

Corresponding Author:

F. Colbourne

Email: [fcolbour@ualberta.ca](mailto:fcolbour@ualberta.ca)

Author(s) ORCID:

A.C.J. Kalisvaart: 0000-0001-6403-1808

A. H. Abrahart: **0000-0003-1452-2427**

F. Colbourne: 0000-0002-9567-2082

**Supplementary Material 1 Index:**

- 1. – Experiment 1 Historical Comparison, Cortical Thickness (Figure 1).

1.2- Experiment 2 DIICP/RICP Event Data (Table 1).

1.3- Experiment 2 DIICP/RICP Event Code

**
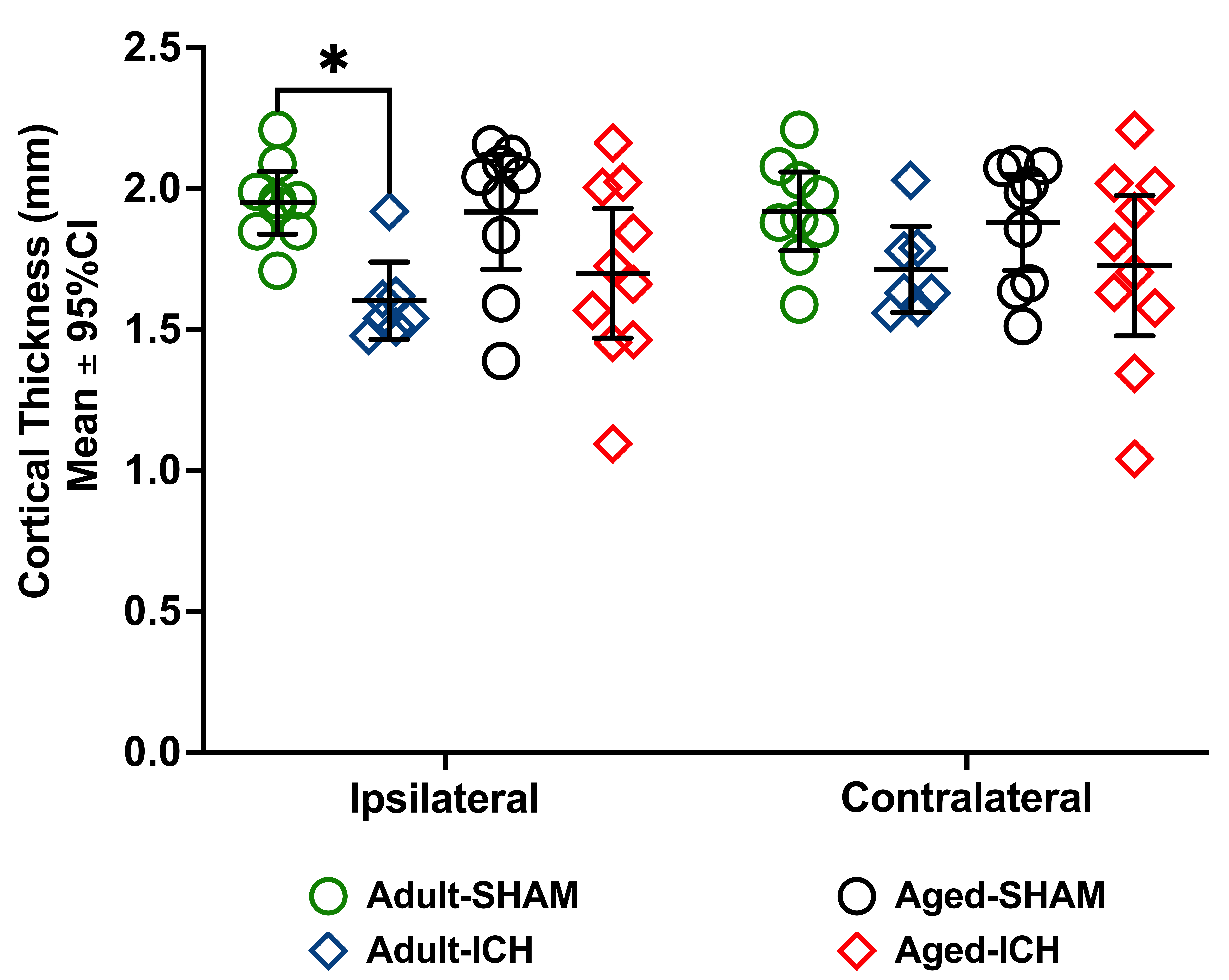
**

**Supplementary Material 1, Figure 1.** Cortical thickness of each hemisphere (ipsilateral-left, contralateral-right) across young adult and aged animals. Only Adult-ICH animals demonstrated a significantly lower cortical thickness ipsilaterally vs. Adult-SHAMs. *p<0.05 vs. Adult-SHAMs.

**Table 1.** DIICP and RICP events, event timing and duration, along with average and maximum ICP over each event across Aged-ICH and Aged-SHAM groups.

| **Animal/Group** | **DIICP Event (s)?** | **Event Timing + Duration** | **Avg. & Max ICP over DIICP Event** | **RICP Events (?)** | **Event Timing + Duration** | **Avg. & Max ICP over RICP Event** |
| --- | --- | --- | --- | --- | --- | --- |
| Animal C  (Aged-SHAM) | DIICP Event #1 | Minute 1254 for 4 minutes | Avg. ICP of 23.31 mmHg  Peak ICP of 24.92 mmHg | N/A | N/A | N/A |
| Animal D  (Aged-ICH) | DIICP Event #1 | Minute 1164 for 5 minutes | Avg. ICP of 28.03 mmHg  Peak ICP of 29.05 mmHg | RICP Event #1 | Minute 1053 for 47 minutes | Avg. ICP of 24.88 mmHg  Peak ICP of 31.36 mmHg |
|  | DIICP Event #2 | Minute 1307 for 7 minutes | Avg. ICP of 30.71 mmHg  Peak ICP of 35.48 mmHg |  |  |  |
|  | DIICP Event #3 | Minute 1365 for 5 minutes | Avg. ICP of 23.57 mmHg Peak ICP of 24.75 mmHg | RICP Event #2 | Minute 1183 for 69 minutes | Avg. ICP of 26.91 mmHg  Peak ICP of 29.58 mmHg |
|  | DIICP Event #4 | Minute 1403 for 5 minutes | Avg. ICP of 22.37 mmHg  Peak ICP of 23.20 mmHg |  |  |  |
| Animal G  (Aged-ICH) | N/A | N/A | N/A | RICP Event #1 | Minute 1276 for 16 minutes | Avg. ICP of 23.12 mmHg  Peak ICP of 29.82 mmHg |
|  |  |  |  | RICP Event #2 | Minute 1294 for 13 minutes | Avg. ICP of 21.57 mmHg  Peak ICP of 22.83 |
|  |  |  |  | RICP Event #3 | Minute 1319 for 6 minutes | Avg. ICP of 21.37 mmHg  Peak ICP of 22.82 mmHg |
|  |  |  |  | RICP Event #4 | Minute 1337 for 19 minutes | Avg. ICP of 20.89 mmHg  Peak ICP of 25.09 mmHg |
|  |  |  |  | RICP Event #5 | Minute 1350 for 10 minutes | Avg. ICP of 22.22 mmHg  Peak ICP of 26.11 mmHg |
|  |  |  |  | RICP Event #6 | Minute 1380 for 5 minutes | Avg. ICP of 22.14 mmHg  Peak ICP of 24.45 mmHg |
|  |  |  |  | RICP Event #7 | Minute 1389 for 17 minutes | Avg. ICP of 21.91 mmHg  Peak ICP of 27.43 mmHg |
|  |  |  |  | RICP Event #8 | Minute 1401 for 14 minutes | Avg. ICP of 21.26 mmHg  Peak ICP of 23.10 mmHg |
|  |  |  |  | RICP Event #9 | Minute 1422 for 3 minutes | Avg. ICP of 20.72 mmHg  Peak ICP of 21.10 mmHg |
| Animal H  (Aged-SHAM) | DIICP Event #1 | Minute 406 for 4 minutes | Avg. ICP of 15.52 mmHg  Peak ICP of 15.95 mmHg | N/A | N/A | N/A |
| Animal I  (Aged-ICH) | DIICP Event #1 | Minute 148 for 3 minutes | Avg. ICP of 33.62 mmHg  Peak ICP of 35.86 mmHg | N/A | N/A | N/A |
|  | DIICP Event #2 | Minute 261 for 3 minutes | Avg. ICP of 46.7 mmHg  Peak ICP of 49.33 mmHg |  |  |  |
|  | DIICP Event #3 | Minute 315 for 3 minutes | Avg. ICP of 61.70 mmHg  Peak ICP of 65.84 |  |  |  |
| Animal O  (Aged-ICH) | N/A | N/A | N/A | RICP Event #1 | Minute 1188 for 49 minutes | Avg. ICP of 21.93 mmHg  Peak ICP of 23.31 mmHg |
|  | N/A | N/A | N/A | RICP Event #2 | Minute 1245 for 67 minutes | Avg. ICP of 21.36 mmHg  Peak ICP of 22.53 mmHg |
|  | N/A | N/A | N/A | RICP Event #3 | Minute 1313 for 25 minutes | Avg. ICP of 21.71 mmHg  Peak ICP of 22.92 mmHg |
| Animal S  (Aged-ICH) | DIICP Event #1 | Minute 158 for 3 minutes | Avg. ICP of 20.97 mmHg  Peak ICP of 21.41 mmHg | RICP Event #1 | Minute 234 for 120 minutes | Avg. ICP of 28.61 mmHg  Peak ICP of 33.84 mmHg |
|  | N/A | N/A | N/A | RICP Event #2 | Minute 355 for 6 minutes | Avg. ICP of 24.27 mmHg  Peak ICP of 32.19 mmHg |

**Supplementary Material 1.3. Experiment 2 DIICP/RICP Code**

import pandas as pd

import xlwt

from xlwt import Workbook

import numpy as np

#THIS PROGRAM OUTPUTS DIICP EVENTS

#LOAD IN DATAFILE

print("Before you start, make sure you have this file saved in the same folder as the data/excel file you'd like to work with. Otherwise it won't work.")

print("Next, load in the datafile. If it is an excel file (.xlsx) make sure you save as a .csv file, then input that filename when prompted.")

file_name = input("What is the filename (include .csv at end): ")

data = pd.read_csv(file_name)

### CHECKS ###

# ## if you remove the # from the below checks, you can check if the data is all there (rows and columns)

# print(data)

# row1 = pd.DataFrame(data)

# print(row1)

#This function returns a list of all of the rat letters: type <str> [list]

def letters_in_list(N):

rat_letter_list = []

for col in data.columns[1:N+1]:

rat_letter_list.append(col)

return rat_letter_list

#This function takes in the rat letter and returns all of the datapoints for that rat in a list

def ind_rat_1440(rat_letter):

temp = pd.Series(data[rat_letter])

list_1440 = list(temp)

return list_1440

#This function combines all of the individual rat_1440 lists into a list of lists

def all_rat_1440(rat_letter_list):

list_1440 = []

for rat in rat_letter_list:

ind_list = ind_rat_1440(rat)

list_1440.append(ind_list)

return list_1440

#This function returns a list of the average magnitude of ICP spikes above 22mmHg across animals

def avg_above_20(rat):

points = ind_rat_1440(rat)

total = 0

small = True

size = len(points)

used_size = 0

for i in range(0, size):

if pd.isna(points[i]) == True:

i += 1

continue

if points[i] >= 20.0:

small = False

used_size += 1

total += points[i]

if small == False:

avg = (total/used_size)

elif small == True:

avg = 'non-existent'

return avg

#This function takes in the 1440 data for one rat and returns a list of corresponding moving averages

def moving_average(rat_data, n):

movavg_list = []

copy_data = [] #copy_data is so that the raw data isn't altered

for d in rat_data:

copy_data.append(d)

max = 60

for i in range(0, n):

if i == 0:

mov_avg = copy_data[0]

movavg_list.append(mov_avg)

i += 1

elif pd.isna(copy_data[i]) == True:

copy_data[i] = mov_avg

movavg_list.append(mov_avg)

i += 1

elif i <= max:

current_window = copy_data[0:i]

mov_avg = sum(current_window)/i

movavg_list.append(mov_avg)

i += 1

elif i > max:

current_window = copy_data[i-max:i]

mov_avg = sum(current_window)/max

movavg_list.append(mov_avg)

i += 1

return movavg_list

#This function combines all of the individual moving average lists into a list of lists

def all_moving_avg(major_1440_list, n):

move_1440 = []

for data in major_1440_list:

ind_list = moving_average(data, n)

move_1440.append(ind_list)

return move_1440

#This function takes in a rat's current ICP data and mov avg data, and totalvalues given for 1 list (n) and compares it

#returns: a list with number of events (int), length of each event (int list), and start time for each event (int list)

def diicp_per_rat(cur_ICP, mov_avg, n):

diicp_event = False

length_event = 0

event_nums = 0

length_event_list = []

flag_list = []

for i in range(0, n-1):

if pd.isna(cur_ICP[i]) == True:

continue

else:

if cur_ICP[i] >= (mov_avg[i] + 10.0): #conditions for event are met

if diicp_event == False: #set up like this because when diicp_event is still false in this loop, we're at the start of a new diicp_event

flag = i+1 #minute DIICP occurred

diicp_event = True

if diicp_event == True:

length_event += 1

i += 1

elif pd.isna(cur_ICP[i]) == True:

if length_event > 3:

event_nums += 1

flag_list.append(flag)

length_event_list.append(length_event)

length_event = 0

diicp_event = False

else: #conditions for event not met

if length_event >= 3:

event_nums += 1

flag_list.append(flag)

length_event_list.append(length_event)

length_event = 0

diicp_event = False

i += 1

#this block of code combines 2 events if they are 3 minutes or less apart

base_nums = event_nums

for j in range(0, event_nums-3):

if (j+1 >= len(flag_list)):

break

else:

start = (flag_list[j] + length_event_list[j])

stop = (flag_list[j+1])

difference = start - stop

if abs(difference) <= 3:

event_nums -= 1

length_event_list[j] += length_event_list[j+1]

delete = flag_list[j+1]

flag_list.remove(delete)

length_event_list.remove(length_event_list[j+1])

return[event_nums, length_event_list, flag_list]

#this function is basically our output. It returns a list of all of the lines that will be read into a text file in another function

def all_diicp_data(major_1440, major_move, N, n, rat_letter_list):

line_list = []

count = 0

for rat in rat_letter_list:

num = rat_letter_list.index(rat)

avg = str(avg_above_20(rat))

if (avg[0] != 'n'):

avg += 'mmHg'

data = diicp_per_rat(major_1440[num], major_move[num], n)

if data[0] == 0:

data = "No DIICP events occurred for this animal"

datadata = str(rat+': '+data+'. The avg ICP above 20mmHg is '+avg)

line_list.append(datadata)

else:

datadata = str(rat)+': '+str(data[0])+' event(s) occurred. The avg ICP above 20mmHg is '+avg

line_list.append(datadata)

for i in range(data[0]):

count += 1

datadata = ' Sheet #'+str(count)+': Ocurred at minute '+str(data[2][i])+' for '+str(data[1][i])+'mins.'

line_list.append(datadata)

return line_list

#This function returns the event information for each rat that had diicp events in a list

def data_for_sheets(major_1440, major_move, N, n, rat_letter_list):

sheets_list = []

for rat in rat_letter_list:

num = rat_letter_list.index(rat)

data = diicp_per_rat(major_1440[num], major_move[num], n)

if data[0] != 0:

sheets_list.append([rat,data])

return sheets_list

#This function takes in the list computed in the previous function and turns it into a dictionary with rat_letter: [diicp outputs] pairs

def data_to_dictionary(sheets_data):

animal_letters = []

animal_list = []

for animal in sheets_data:

animal_letters.append(animal[0])

animal_list.append(animal[1])

animal_dictionary = dict(zip(animal_letters, animal_list))

return animal_dictionary

#This function takes in the letters of all the rats that had diicp events and the dictionary made in the function above

#it returns a list of length all events, with each element being a list of the previous 60 data points for each event in alphabetical and then temporal order

def all_previous_60(sheet_letters, sheets_dictionary):

export_list = []

data = ind_rat_1440(sheet_letters)

info = sheets_dictionary[sheet_letters]

for i in range(info[0]):

start = info[2][i]

if start >= 60:

export = data[(start-60):start]

else:

export = data[0:start]

export_list.append(export)

return export_list

#This function takes in the letters of all the rats that had diicp events as well as the dictionary containing all of the event information.

#it returns an int value of the total number of diicp events found in the dataset.

def number_of_events(sheet_letters, sheets_dictionary):

count = 0

for letter in sheet_letters:

data = sheets_dictionary[letter]

event_num = data[0]

count += event_num

return count

#This function takes in the list of all of the sets of 60 datapoints and creates an excel file for each of them

#This excel file will be saved to the location that you have python_diicp.py(this file) and the excel file you inputted at the beginning (they must be saved in the same folder)

def sheet_exports(sheet_letters, sheets_dictionary):

wb = xlwt.Workbook()

maxx = 60

for letter in sheet_letters:

ws = wb.add_sheet(letter)

ws.write(0, 0, 'TIME')

start_stats = 62

ws.write(start_stats, 0, "no. of events")

ws.write(start_stats+1, 0, 'start(MIN)')

ws.write(start_stats+2, 0, 'duration(MIN)')

for t in range(maxx):

ws.write(t+1, 0, str(t+1)+' MIN')

row = 1

name = letter

data = sheets_dictionary[letter]

event_nums = data[0] #int

flag_list = data[2] #list

length_list = data[1] #list

previous_60 = all_previous_60(letter, sheets_dictionary) #listoflist

ws.write(start_stats, 1, event_nums)

for i in range(event_nums):

ws.write(start_stats+1, row, flag_list[i])

ws.write(start_stats+2, row, length_list[i])

ws.write(0, row, (str(name)+' '+str(i+1)))

size = len(previous_60[i])

if size < maxx:

missing = (maxx - size)

for m in range(0, missing):

ws.write(m+1, row, np.nan)

for n in range(missing, 60):

ws.write(n+1, row, float(previous_60[i][n-missing]))

else:

for j in range(60):

ws.write(j+1, row, float(previous_60[i][j]))

row += 1

wb.save('diicp_'+str(letter)+'.xls')

#This function takes in our output from all_diicp_data() and creates a text file with our output that will also be saved in the same location that you have this file saved

def text_exports(all_data):

new_data = []

for line in all_data:

if line[0] == 'm':

line = ' '+line

new_data.append(line)

with open('diicp_outputs.txt', 'w') as f:

for line in new_data:

f.write(line)

f.write('\n')

f.close()

def main():

N = len(data.columns)-1 #should be 20 if there are 20 rats

n = len(data) #should be 1440 for 1440 minutes

#print(N); print(n)

rat_letter_list = letters_in_list(N) #this of all the rat letters. Ex) 'A'

major_1440_list = all_rat_1440(rat_letter_list) #list of all of the current_ICP data for each rat

major_move_list = all_moving_avg(major_1440_list, n) #list of all of the associated moving average/baseline data for each rat

all_data = all_diicp_data(major_1440_list, major_move_list, N, n, rat_letter_list) #this is a list of the data for all diicp events that occurred

sheets_data = data_for_sheets(major_1440_list, major_move_list, N, n, rat_letter_list) #this is the data in list form for each diicp event

sheets_dictionary = data_to_dictionary(sheets_data) #this is the dictionary form of sheets_data in rat:data key value pairs

#This block of code creates a list called sheet_letters of all the rats that had diicp events occur in alphabetical order

sheet_letters = []

sheet_letters_temp = sheets_dictionary.keys()

for i in sheet_letters_temp:

sheet_letters.append(i)

total_events = number_of_events(sheet_letters, sheets_dictionary)

print(str(total_events)+' diicp events found.')

sheet_exports(sheet_letters, sheets_dictionary) #creates a spreadsheet for each diicp event

text_exports(all_data) #creates a textfile with information about the data's diicp events

print("Success, go check the folder this file is saved in to find the event spreadsheets and output text file.")

main()

import pandas as pd

import xlwt

from xlwt import Workbook

import numpy as np

#THIS PROGRAM OUTPUTS RICP EVENTS

#LOAD IN DATAFILE

print("Before you start, make sure you have this file saved in the same folder as the data/excel file you'd like to work with. Otherwise it won't work.")

print("Next, load in the datafile. If it is an excel file (.xlsx) make sure you save as a .csv file, then input that filename when prompted.")

file_name = input("What is the filename (include .csv at end): ")

data = pd.read_csv(file_name)

### CHECKS ###

# ## if you remove the # from the below checks, you can check if the data is all there (rows and columns)

# print(data)

# row1 = pd.DataFrame(data)

# print(row1)

# ##check: correctly has 1440 rows and 21 columns

#This function returns a list of all of the rat letters: type <str> [list]

def letters_in_list(N):

rat_letter_list = []

for col in data.columns[1:N+1]:

rat_letter_list.append(col)

return rat_letter_list

#This function takes in the rat letter and returns all of the datapoints for that rat in a list

def ind_rat_1440(rat_letter):

temp = pd.Series(data[rat_letter])

list_1440 = list(temp)

return list_1440

#This function combines all of the individual rat_1440 lists into a list of lists

def all_rat_1440(rat_letter_list):

list_1440 = []

for rat in rat_letter_list:

ind_list = ind_rat_1440(rat)

list_1440.append(ind_list)

return list_1440

#This function returns a list of the average magnitude of ICP spikes above 22mmHg across animals

def avg_above_20(rat):

points = ind_rat_1440(rat)

total = 0

small = True

size = len(points)

used_size = 0

for i in range(0, size):

if pd.isna(points[i]) == True:

i += 1

continue

if points[i] >= 20.0:

small = False

used_size += 1

total += points[i]

if small == False:

avg = (total/used_size)

elif small == True:

avg = 'non-existent'

return avg

#This function takes in the 1440 data for one rat and returns a list of corresponding moving averages

def moving_average(rat_data, n):

movavg_list = []

copy_data = [] #copy_data is so that the raw data isn't altered

for d in rat_data:

copy_data.append(d)

max = 60

for i in range(0, n):

if i == 0:

mov_avg = copy_data[0]

movavg_list.append(mov_avg)

i += 1

elif pd.isna(copy_data[i]) == True:

copy_data[i] = mov_avg

movavg_list.append(mov_avg)

i += 1

elif i <= max:

current_window = copy_data[0:i]

mov_avg = sum(current_window)/i

movavg_list.append(mov_avg)

i += 1

elif i > max:

current_window = copy_data[i-max:i]

mov_avg = sum(current_window)/max

movavg_list.append(mov_avg)

i += 1

return movavg_list

#This function combines all of the individual moving average lists into a list of lists

def all_moving_avg(major_1440_list, n):

move_1440 = []

for data in major_1440_list:

ind_list = moving_average(data, n)

move_1440.append(ind_list)

return move_1440

#This function takes in a rat's current ICP data and mov avg data and compares it

#returns: a list with number of events (int), length of each event (int list), and start time for each event (int list)

def diicp_per_rat(cur_ICP, mov_avg, n):

diicp_event = False

length_event = 0

event_nums = 0

length_event_list = []

flag_list = []

for i in range(0, n-1):

if pd.isna(cur_ICP[i]) == True:

i += 1

continue

else:

if (cur_ICP[i] > 20.0) and (mov_avg[i] >= 20.0): #conditions for event are met

if diicp_event == False: #set up like this because when diicp_event is still false in this loop, we're at the start of a new diicp_event

flag = (i+1) #minute DIICP occurred

diicp_event = True

if diicp_event == True:

length_event += 1

i += 1

elif pd.isna(cur_ICP[i]) == True: #handles events ending on a null value and adds event to events_list and sets diicp_event to False

if length_event > 3:

event_nums += 1

flag_list.append(flag)

length_event_list.append(length_event)

length_event = 0

diicp_event = False

else: #conditions for event not met, ends event and sets diicp_event to False

if length_event >= 3:

event_nums += 1

flag_list.append(flag)

length_event_list.append(length_event)

length_event = 0

diicp_event = False

i += 1

#this block of code combines 2 events if they are 3 minutes or less apart

base_nums = event_nums

for j in range(0, base_nums-3):

if (j+1 >= len(flag_list)):

break

else:

start = (flag_list[j] + length_event_list[j])

stop = (flag_list[j+1])

difference = start - stop

if abs(difference) <= 3:

event_nums -= 1

length_event_list[j] += length_event_list[j+1]

delete = flag_list[j+1]

flag_list.remove(delete)

length_event_list.remove(length_event_list[j+1])

return[event_nums, length_event_list, flag_list]

#this function is basically our output. It returns a list of all of the lines that will be read into a text file in another function

def all_diicp_data(major_1440, major_move, N, n, rat_letter_list):

line_list = []

count = 0

for rat in rat_letter_list:

num = rat_letter_list.index(rat)

avg = str(avg_above_20(rat))

if (avg[0] != 'n'):

avg += 'mmHg'

data = diicp_per_rat(major_1440[num], major_move[num], n)

if data[0] == 0:

data = "No DIICP events occurred for this animal"

datadata = str(rat+': '+data+'. The avg ICP above 20mmHg is '+avg)

line_list.append(datadata)

else:

datadata = str(rat)+': '+str(data[0])+' event(s) occurred. The avg ICP above 20mmHg is '+avg

line_list.append(datadata)

for i in range(data[0]):

count += 1

datadata = ' Sheet #'+str(count)+': Ocurred at minute '+str(data[2][i])+' for '+str(data[1][i])+'mins.'

line_list.append(datadata)

return line_list

#This function returns the event information for each rat that had diicp events in a list

def data_for_sheets(major_1440, major_move, N, n, rat_letter_list):

sheets_list = []

for rat in rat_letter_list:

num = rat_letter_list.index(rat)

data = diicp_per_rat(major_1440[num], major_move[num], n)

if data[0] != 0:

sheets_list.append([rat,data])

return sheets_list

#This function takes in the list computed in the previous function and turns it into a dictionary with rat_letter: [diicp outputs] pairs

def data_to_dictionary(sheets_data):

animal_letters = []

animal_list = []

for animal in sheets_data:

animal_letters.append(animal[0])

animal_list.append(animal[1])

animal_dictionary = dict(zip(animal_letters, animal_list))

return animal_dictionary

#This function takes in the letters of all the rats that had diicp events and the dictionary made in the function above

#it returns a list of length all events, with each element being a list of the previous 60 data points for each event in alphabetical and then temporal order

def all_previous_60(sheet_letters, sheets_dictionary):

export_list = []

data = ind_rat_1440(sheet_letters)

info = sheets_dictionary[sheet_letters]

for i in range(info[0]):

start = info[2][i]

if start >= 60:

export = data[(start-60):start]

else:

export = data[0:start]

export_list.append(export)

return export_list

#This function takes in the letters of all the rats that had diicp events as well as the dictionary containing all of the event information.

#it returns an int value of the total number of diicp events found in the dataset.

def number_of_events(sheet_letters, sheets_dictionary):

count = 0

for letter in sheet_letters:

data = sheets_dictionary[letter]

event_num = data[0]

count += event_num

return count

#This function takes in the list of all of the sets of 60 datapoints and creates an excel file for each of them

#This excel file will be saved to the location that you have python_diicp.py(this file) and the excel file you inputted at the beginning (they must be saved in the same folder)

def sheet_exports(sheet_letters, sheets_dictionary):

wb = xlwt.Workbook()

maxx = 60

for letter in sheet_letters:

ws = wb.add_sheet(letter)

ws.write(0, 0, 'TIME')

start_stats = 62

ws.write(start_stats, 0, "no. of events")

ws.write(start_stats+1, 0, 'start(MIN)')

ws.write(start_stats+2, 0, 'duration(MIN)')

for t in range(maxx):

ws.write(t+1, 0, str(t+1)+' MIN')

row = 1

name = letter

data = sheets_dictionary[letter]

event_nums = data[0] #int

flag_list = data[2] #list

length_list = data[1] #list

previous_60 = all_previous_60(letter, sheets_dictionary) #listoflist

ws.write(start_stats, 1, event_nums)

for i in range(event_nums):

ws.write(start_stats+1, row, flag_list[i])

ws.write(start_stats+2, row, length_list[i])

ws.write(0, row, (str(name)+' '+str(i+1)))

size = len(previous_60[i])

if size < maxx:

missing = (maxx - size)

for m in range(0, missing):

ws.write(m+1, row, np.nan)

for n in range(missing, 60):

ws.write(n+1, row, float(previous_60[i][n-missing]))

else:

for j in range(60):

ws.write(j+1, row, float(previous_60[i][j]))

row += 1

wb.save('ricp_'+str(letter)+'.xls')

#This function takes in our output from all_diicp_data() and creates a text file with our output that will also be saved in the same location that you have this file saved

def text_exports(all_data):

new_data = []

for line in all_data:

if line[0] == 'm':

line = ' '+line

new_data.append(line)

with open('ricp_outputs.txt', 'w') as f:

for line in new_data:

f.write(line)

f.write('\n')

f.close()

def main():

N = len(data.columns)-1 #should be 20 if there are 20 rats

n = len(data) #should be 1440 for 1440 minutes

#print(N); print(n)

rat_letter_list = letters_in_list(N) #this of all the rat letters. Ex) 'A'

major_1440_list = all_rat_1440(rat_letter_list) #list of all of the current_ICP data for each rat

major_move_list = all_moving_avg(major_1440_list, n) #list of all of the associated moving average/baseline data for each rat

all_data = all_diicp_data(major_1440_list, major_move_list, N, n, rat_letter_list) #this is a list of the data for all diicp events that occurred

sheets_data = data_for_sheets(major_1440_list, major_move_list, N, n, rat_letter_list) #this is the data in list form for each diicp event

sheets_dictionary = data_to_dictionary(sheets_data) #this is the dictionary form of sheets_data in rat:data key value pairs

#This block of code creates a list called sheet_letters of all the rats that had diicp events occur in alphabetical order

sheet_letters = []

sheet_letters_temp = sheets_dictionary.keys()

for i in sheet_letters_temp:

sheet_letters.append(i)

total_events = number_of_events(sheet_letters, sheets_dictionary)

print(str(total_events)+' diicp events found.')

sheet_exports(sheet_letters, sheets_dictionary) #creates a spreadsheet for each diicp event

text_exports(all_data) #creates a textfile with information about the data's diicp events

print("Success, go check the folder this file is saved in to find the event spreadsheets and output text file.")

main()
